# Treatment of achalasia with peroral endoscopic myotomy in situs inversus totalis

**DOI:** 10.1002/deo2.49

**Published:** 2021-09-20

**Authors:** Mary Raina Angeli Fujiyoshi, Yuto Shimamura, Yusuke Fujiyoshi, Kei Ushikubo, Yuki Shibata, Yohei Nishikawa, Masashi Ono, Haruo Ikeda, Manabu Onimaru, Haruhiro Inoue

**Affiliations:** ^1^ Digestive Diseases Center Showa University Koto Toyosu Hospital Tokyo Japan

**Keywords:** achalasia, peroral endoscopic myotomy, POEM, situs inversus totalis

## Abstract

Peroral endoscopic myotomy (POEM) has become established as a safe, effective, and versatile minimally invasive endoscopic treatment for achalasia and other esophageal motility disorders. Situs inversus totalis is a rare congenital disorder characterized by a completely reversed position (mirror‐image) of the thoracic and abdominal visceral organs. This case report demonstrated a successful treatment of achalasia in a situs inversus totalis by POEM.

Similar to the POEM procedure in a normal patient, it is important to maintain the orientation throughout the submucosal tunneling while keeping in mind the reversed orientation and anatomical landmarks. The submucosal tunnel and myotomy were created by an anterior approach which is in this case located at the reversed axis, at 10 o'clock position. There were no major technical modifications needed to be carried out by the operator. No adverse events were noted. Improvement in the Eckardt Symptom Score as well as the barium esophagogram and high‐resolution manometry findings on 2‐month follow‐up exhibited that although POEM was performed in a reversed orientation, similar effects and outcomes were achieved, indicating a successful procedure in this case.

In summary, by keeping in mind the reversed positioning and anatomical landmarks in situs inversus totalis, POEM shows to be a safe, effective, and versatile intervention in treating achalasia in situs inversus totalis without the need for major modifications in the procedural technique.

## INTRODUCTION

More than a decade after the first case of peroral endoscopic myotomy (POEM) in a human in 2008, POEM has become established as a safe and effective minimally invasive endoscopic treatment for achalasia and other esophageal motility disorders,^1^ with efficacy rates of more than 95%.^2^ To date, more than 2300 cases have been performed at our institution (Showa University, Japan), representing the largest referral center for POEM in Japan. By making small modifications to the technique (adjusting the myotomy length, choosing the orientation of the tunnel etc.), POEM has proven to be a versatile intervention for achalasia and other esophageal motility disorders.

Situs inversus totalis is a rare congenital disorder characterized by a completely reversed position (mirror‐image) of the thoracic and abdominal visceral organs, occurring in 1/4100–8000 live births.^3^, ^4^

Herein, we report a case of a patient examined at our institution and diagnosed with achalasia and situs inversus totalis, treated successfully with POEM.

## CASE REPORT

This is a case of a 39‐year‐old Japanese man, otherwise healthy, who presented with a 5‐year history of occasional, tolerable chest pain and 2‐year history of dysphagia and food impaction. Symptoms gradually progressed and started affecting his daily activities since 1 year prior to the consultation. Patient also experienced unintentional 5–10 kg of weight loss over the past year. No previous consultation and treatment were noted. Eckardt Symptom Score (ESS) was 6, and Vaezi score was 8. The patient underwent routine evaluation for achalasia which includes upper gastrointestinal endoscopy, barium esophagogram, high‐resolution manometry (HRM), and computed tomography (CT) scan of the thorax and upper abdomen.

**FIGURE 1:**
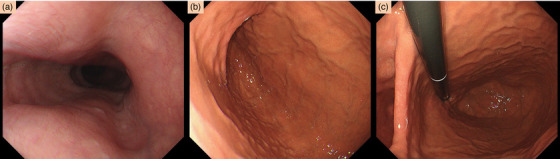
**Endoscopic findings**: Preoperative endoscopic findings showing reversed anatomical landmarks such as the left main bronchus impression (a). In this case, it became the right main bronchus compression. The orientation of the gastric folds (b) and the retroflexed view (c) are also reversed

**FIGURE 2:**
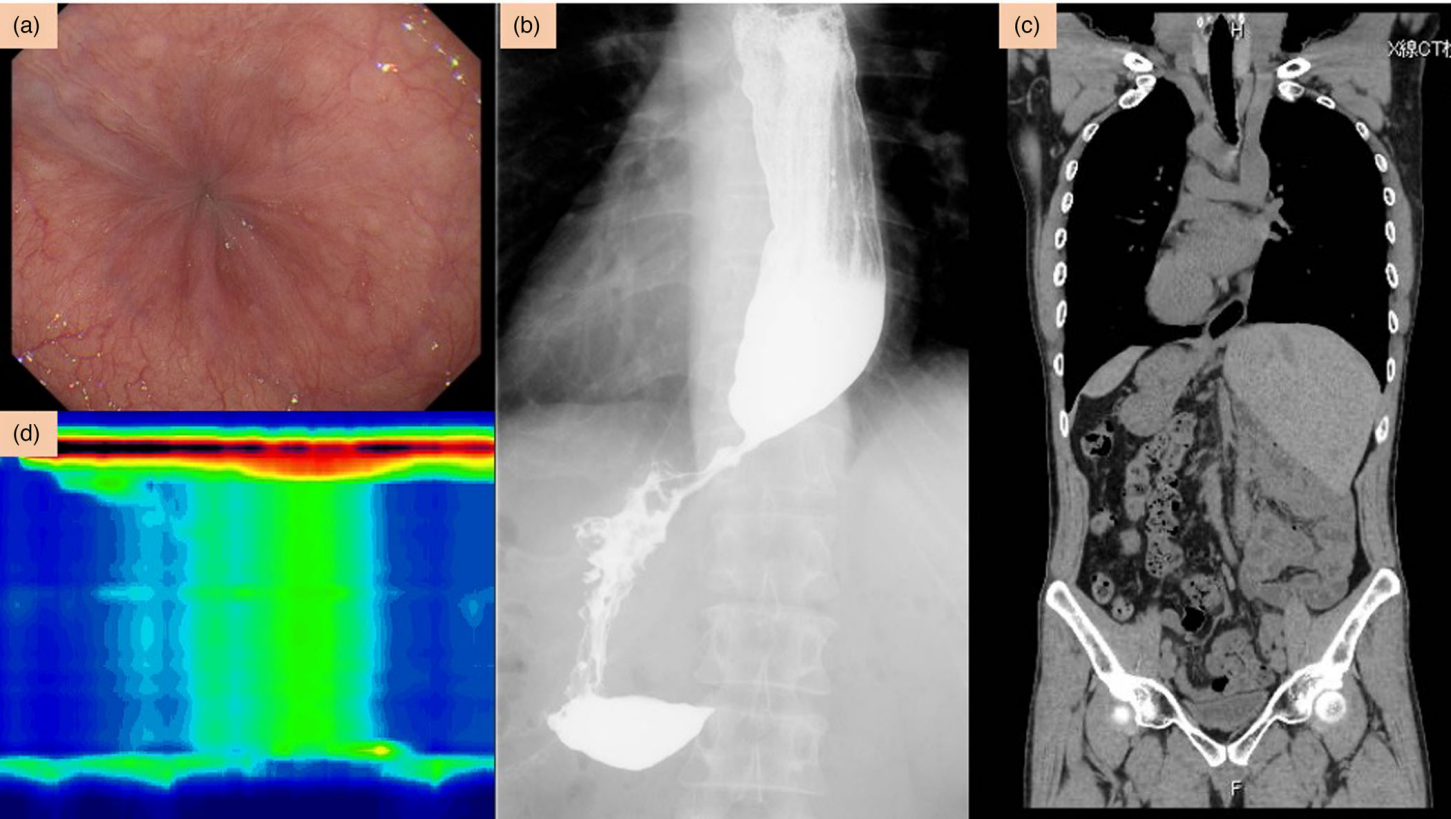
**Preoperative evaluation**: (a) Endoscopic finding showing a resistance in passing through the gastroesophageal junction (GEJ) as well as the rosette sign. (b) Barium esophagogram reveals a delay in the passage of the contrast and a narrowing at the lower esophageal sphincter (LES), creating the classic “bird's beak sign.” Noticeably, the barium esophagogram findings seemed to show an inverted (mirror‐image) positioning of the visceral organs. (c) CT scan shows the completely reversed position of the thoracic and abdominal visceral organs, establishing the diagnosis of situs inversus totalis. (d) High‐resolution manometry findings are consistent with Type II achalasia, showing panpressurization.

Endoscopic examination was remarkable for reversed anatomical landmarks such as the left main bronchus compression and appearance of the gastric folds (Figure 1). In addition, endoscopic examination findings also showed a resistance in passing through the gastroesophageal junction (GEJ) as well as the rosette sign^5^ (Figure 2a). On barium esophagogram, delay in the passage of the contrast and a narrowing at the lower esophageal sphincter, creating the classic “bird's beak sign” were observed (Figure 2b). Noticeably, the barium esophagogram findings seemed to show an inverted (mirror‐image) positioning of the thoracic and abdominal organs. CT scan confirmed the completely reversed position of the thoracic and abdominal visceral organs (Figure 2c), establishing the diagnosis of situs inversus totalis. HRM findings were consistent with Type II achalasia, showing panpressurization^6^ (Figure 2d). Median integrated relaxation pressure (IRP) was 31.5 mm Hg. The diagnosis was discussed with the patient, and POEM was decided to be performed. This procedure has been approved by the ethics committee of Showa University Koto Toyosu Hospital and was performed in accordance with the Declaration of Helsinki. Written informed consent was obtained from the patient prior to the procedure.

**FIGURE 3:**
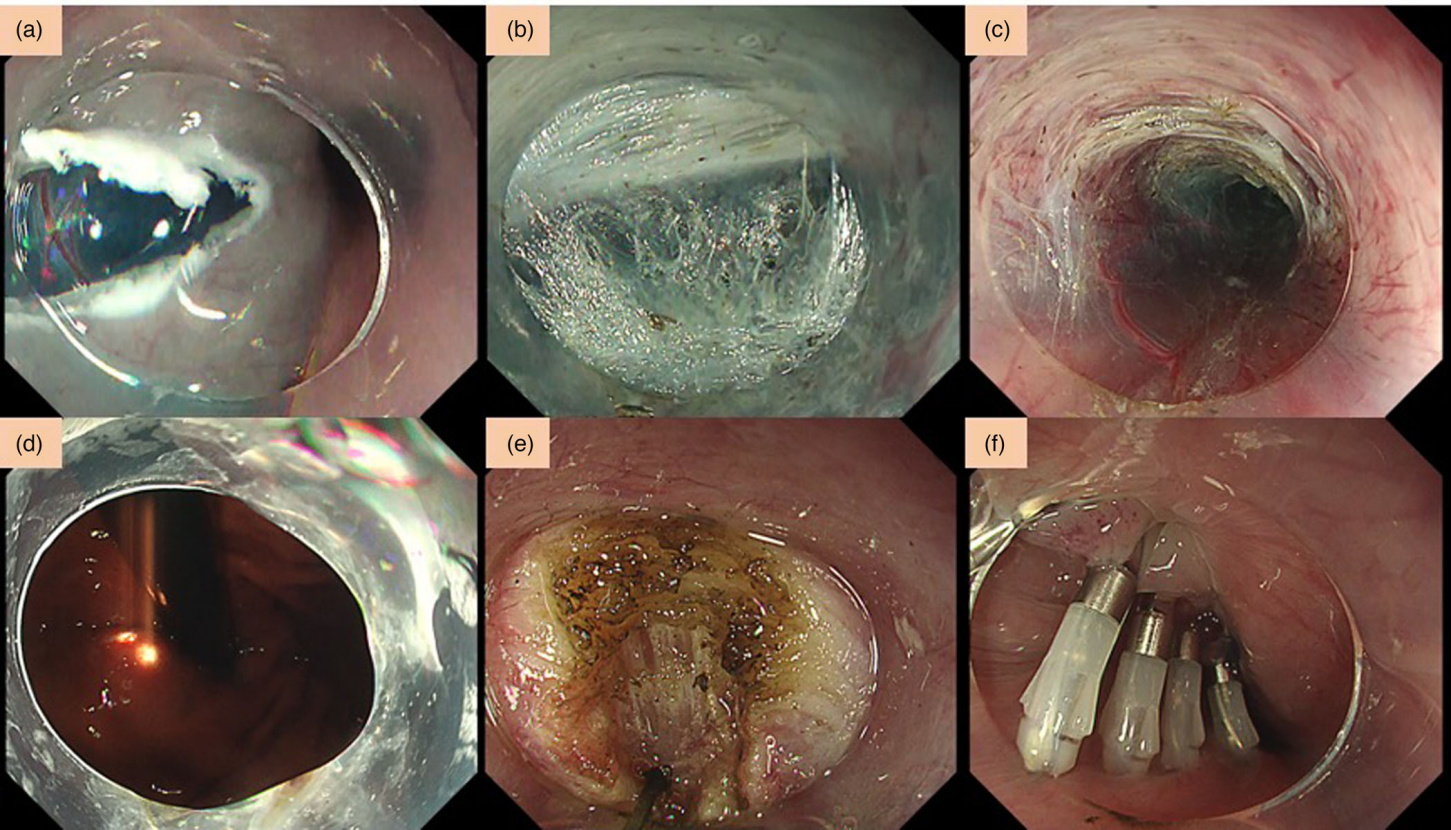
**POEM technique**: (a) Mucosal incision was done by an anterior approach, which is, in this case, at the 10 o'clock position. (b and c) A submucosal tunnel was created from the esophageal side, passing through the GEJ, and 3 cm into the gastric side. (d) The length of the submucosal tunnel was confirmed by double scope method. (e) A 10 cm selective circular myotomy was performed. (f) Closure of the mucosal entry site was done with the use of hemostatic endoclips

POEM was performed under general anesthesia, with the use of a single‐channel therapeutic endoscope (GIF‐H290T; Olympus Medical Corp., Tokyo, Japan). A super soft hood (Space Adjuster, TOP Corp., Tokyo, Japan)^7^ was used as a distal attachment. For mucosal incision, submucosal dissection, and myotomy, a triangle‐tip knife with water jet function (TriangleTipKnifeJ KD‐645; Olympus) was used. Mucosal incision was done by an anterior approach, which is, in this case, at the 10 o'clock position (Figure 3a). A submucosal tunnel (Figure 3b,c) was created from the esophageal side, passing through the GEJ, and 3 cm into the gastric side. The length of the submucosal tunnel was confirmed by double scope method (Figure 3d). A 10 cm selective circular myotomy was performed (Figure 3e). Closure of the mucosal entry site was done with the use of hemostatic endoclips (Figure 3f). Overall procedure time was 68 min, and no adverse events were noted.

On 2‐month follow‐up, patient's ESS improved to 2. Barium esophagogram showed a marked improvement in the passage of the contrast. HRM findings revealed a decrease in the median IRP (15.4 mm Hg).

## DISCUSSION

In this case report, the experience drawn from the pioneer institution of POEM on the treatment of achalasia with POEM in a situs inversus totalis patient is presented. To our knowledge, this is the first case report in Japan and only the second worldwide.^8^

**FIGURE 4:**
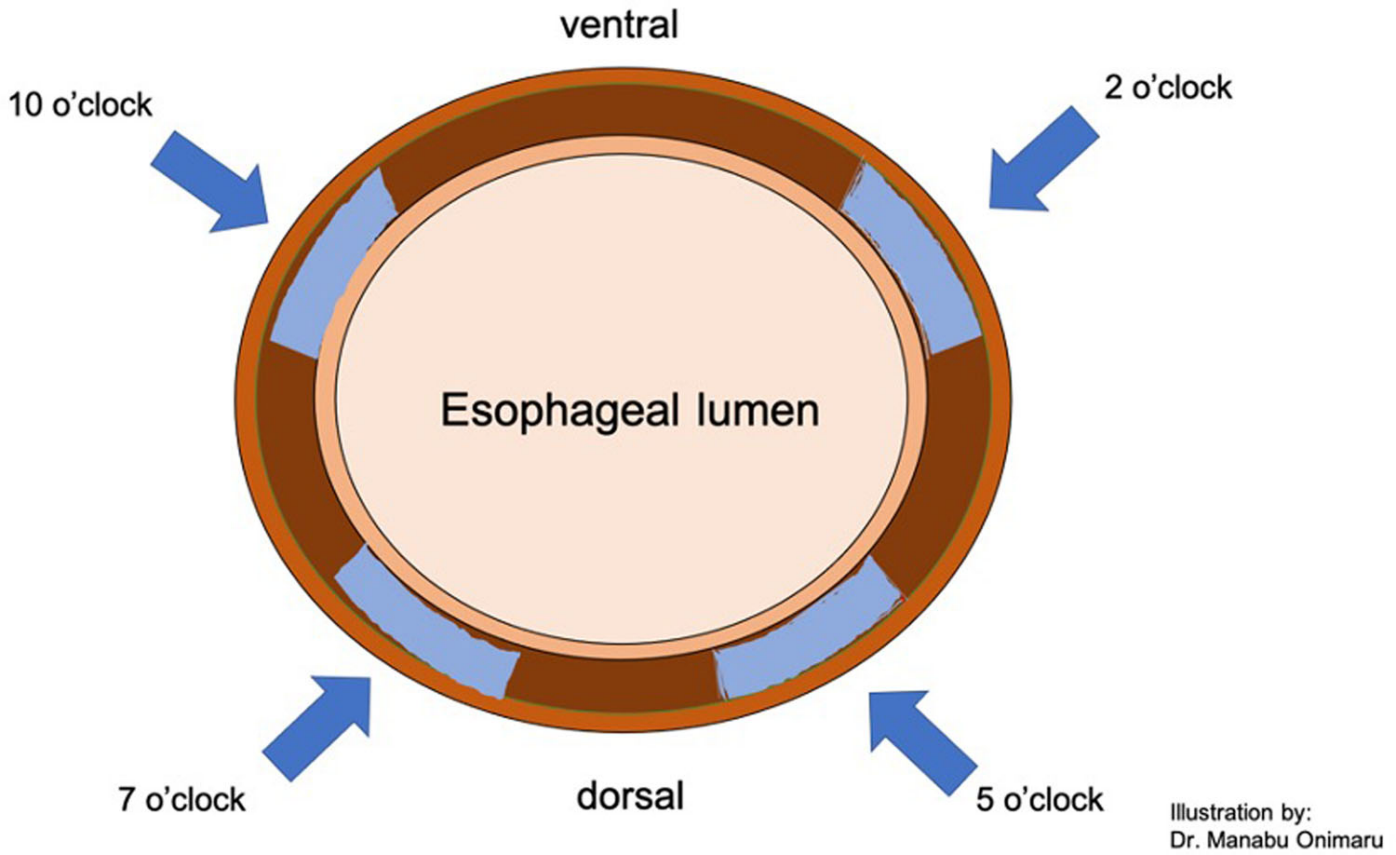
**Illustration of the esophageal wall and the different axis approaches in POEM**: In normal patients, the anterior approach is at 2 o'clock position leading straight to the lesser curvature in the gastric side, while the posterior approach is at 5 o'clock. However, in a situs inversus totalis patient, these anatomical landmarks would be reversed; hence, the anterior approach would be at 10 o'clock and posterior at 7 o'clock

One critical factor in attaining a successful POEM procedure is ensuring the correct orientation of the submucosal tunnel. In normal patients, with the left main bronchus compression maintained at 12 o'clock position, the anterior approach is at 2 o'clock position leading straight to the lesser curvature in the gastric side, while the posterior approach is at 5 o'clock. However, in a situs inversus totalis patient, these anatomical landmarks would be reversed; hence, the anterior approach would be at 10 o'clock and posterior at 7 o'clock (Figure 4). This 10 o'clock position would be the axis leading straight to the lesser curvature in the gastric side in situs inversus totalis; hence, for this case, the anterior approach was chosen. Similar to the POEM procedure in a normal patient, it is important to maintain the orientation throughout the submucosal tunneling while keeping in mind the reversed orientation and anatomical landmarks. If the submucosal tunnel is not straight due to deviation from the original orientation or axis, the odds of an incomplete myotomy increase.

An inadequate or incomplete myotomy on the gastric side is the main risk factor for clinical failure of POEM. Confirmation of the myotomy length must be performed with the double scope method along with identification of the palisade vessels in the esophageal side and submucosal spindle veins in the gastric side.^9^, ^10^ During the double‐scope method, one endoscope is inserted into the submucosal tunnel then subsequently observing its transillumination with the second endoscope in retroflexed view of the gastric cardia.

As demonstrated in this case report, the operator created the submucosal tunnel and myotomy by an anterior approach, at the 10 o'clock position since the anterior approach is the axis that leads straight to the lesser curvature in the gastric side. Other than changing the orientation and performing the procedure in an inverted visual field, there were no major technical modifications needed to be carried out by the operator. The overall procedural time was comparable to that of the average time in our institution.^10^ Improvement in the ESS as well as the barium esophagogram and HRM findings on 2‐month follow‐up exhibited that although POEM was performed in a reversed orientation, similar effects and outcomes were achieved, indicating a successful procedure in this case.

In summary, by keeping in mind the reversed positioning and anatomical landmarks in situs inversus totalis, POEM shows to be a safe, effective, and versatile intervention in treating achalasia in situs inversus totalis without the need for major modifications in the procedural technique.

## CONFLICT OF INTERESTS

Haruhiro Inoue is an advisor of Olympus Corporation and Top Corporation. He has also received educational grants from Olympus Corp., and Takeda Pharmaceutical Co. All other authors have no conflict of interests to declare.

## FUNDING INFORMATION

None.
